# The Utility and Safety of Prophylactic Tranexamic Acid in Tonsillectomy: A Systematic Review and Meta‐analysis

**DOI:** 10.1002/ohn.973

**Published:** 2024-10-01

**Authors:** Hussein Smaily, Patrick Cherfane

**Affiliations:** ^1^ Department of Otolaryngology–Head and Neck Surgery Laval University Quebec Canada; ^2^ Centre Hospitalier de Versailles, Hôpital André Mignot Le Chesnay‐Rocquencourt France

**Keywords:** hemorrhage, systematic review, tonsillectomy, tranexamic acid

## Abstract

**Objective:**

We conducted a systematic review of randomized controlled trials (RCTs) to evaluate the efficacy of tranexamic acid (TXA) in reducing posttonsillectomy hemorrhage (PTH).

**Data Sources:**

We searched MEDLINE, EMBASE, and CENTRAL for RCT comparing prophylactic TXA to control in patients undergoing tonsillectomy.

**Review Methods:**

Per Preferred Reporting Items for Systematic Review and Meta‐analysis guidelines, the databases were searched from date of inception through October 2023. RCTs of patients undergoing tonsillectomy or adenotonsillectomy and receiving prophylactic TXA versus control were included. Two reviewers screened citations, extracted data, assessed the risk of bias, and classification of Grading of Recommendations, Assessment, Development, and Evaluation independently. Standardized mean difference with 95% confidence interval (CI) was applied for continuous variables. Dichotomous data were expressed as relative risk with 95% CI.

**Results:**

A total of 10 RCT were included in our quantitative analysis. Eight studies reported on PTH rate. Prophylactic TXA showed non‐significant decrease in PTH (relative risk or risk ratio [RR]: 0.62 [0.35, 1.10]). Sensitivity analysis showed significant decrease in PTH after exclusion of High‐risk bias studies (RR: 0.48 [0.30, 0.77]). Intraoperative blood loss volume was significantly lower in the TXA group (35.59 mL [−48.19, −22.99]).

**Conclusion:**

Overall, this study showed a tendency toward lesser PTH rate with prophylactic TXA. However, this tendency only reaches statistical significance when studies with high risk of bias are excluded. Well‐designed trials are still needed to support our observations.

## Rationale

Tonsillectomy is one of the most performed surgical procedures worldwide. It carries however the risk of serious complications such as bleeding, respiratory distress, and even death. Interest in tranexamic acid (TXA) has recently emerged after reports of its role in reducing blood loss and need for transfusion in different surgical fields.^1^ TXA is a synthetic derivative of the amino acid lysine with antifibrinolytic activity by binding plasminogen and inhibiting plasmin and thus stabilizing fibrin clots. A recent systematic review (SR) evaluated the efficacy of TXA after tonsillectomy and showed a significant reduction in posttonsillectomy hemorrhage (PTH) rate.^2^ However, this meta‐analysis (MA) has some methodological gaps that undermine the validity of the results. First, it excluded studies of patients undergoing concomitant adenoidectomy (mainly pediatric patients) and showed conflicting results between adult and pediatric population.^2^ Adenoidectomy is often performed alongside tonsillectomy in children with obstructive sleep apnea syndrome (OSAS), which is the most common surgical indication in the pediatric population.^3^ Adenoidectomy is rarely responsible for troublesome per or postoperative hemorrhage.^4^ Excluding studies that include concomitant adenoidectomy could compromise the robustness of Kuo et al's MA findings by the exclusion of high yield pediatric studies on the subject.^2^ Second, this MA^2^ included nonrandomized controlled trials (RCTs) (mainly retrospective chart review studies) and third it considered blood volume loss as the main outcome which we believe is not the best outcome to consider when judging the clinical and practical efficacy of TXA. Per‐operative troublesome bleeding may occur secondary to technical issues such as disrupting the pharyngeal muscular layer or rupturing an artery (tonsillar bed is highly vascularized with 5 main arteries^5^). A ruptured artery will bleed at high pressure and won't respond to basic coagulation cascade as fast as one might imagine. In 2019, the American Academy of Otolaryngology–Head and Neck Surgery published the updated clinical guidelines practice for tonsillectomy in children.^6^ Despite some promising evidence, TXA was not incorporated in the algorithmic management of patients undergoing tonsillectomy. Hence, we believe that there is still a gap in the literature and there is a specific need for high level evidence on the efficacy of prophylactic TXA in tonsillectomy.

## Objective

The objective of this study is to evaluate the effect of prophylactic TXA versus control or standard of care in patients undergoing tonsillectomy in reducing PTH.

Our secondary outcomes consisted of: Perioperative blood volume loss, need for transfusion, need for further intervention, and adverse effects.

## Materials and Methods

### Design

Our SR and MA were conducted using a defined protocol, in accordance with the Cochrane handbook for SR and MA.^7^ A Population, Intervention, Comparison, Outcome, Timing, and Settings (PICOTS) statement was followed to assess reviewed studies for inclusion and exclusion.^8^ PICOTS criteria are as follows:

- Population: Patients undergoing tonsillectomy.
- Intervention: Administration of TXA (any route) perioperatively.
- Comparison: Administration of placebo/control group.
- Outcome: PTH rate, intraoperative blood loss, need for transfusion and further intervention, adverse effects.
- Timing: Postoperative follow‐up required to measure the primary outcome (PTH).
- Settings: Intervention provided within a hospital setting as a day‐surgery or a short‐stay.

The study protocol was registered in the international prospective register of SR platform PROSPERO (CRD42024530380) and the results were reported according to the Preferred Reporting Items for SR and MA.^9^, ^10^

### Search Strategy

Detailed search strategies were developed for MEDLINE (January 1948 to October 2023), EMBASE (1947 to October 2023), and CENTRAL Databases (Cochrane library until October 2023). A combination of Medical Subject Headings, Emtree words, and broad keywords were used in combination with high sensitivity filters for clinical trials to generate the list of citation.^11^, ^12^ The Search Study used for Medline (PubMed Interface) is illustrated in Supplemental Table S1, available online. All included studies and related SR were searched for any missing relevant publication. We also searched for ongoing and unpublished clinical trials in http://www.clinicaltrials.gov and http://www.controlled-trials.com registries.

### Eligibility Criteria

Inclusion criteria consisted of studies that meet all the following criteria:

(1) Randomized controlled trials.
(2) Patients of all ages undergoing tonsillectomy or adenotonsillectomy (AT).
(3) TXA delivered pre‐ or perioperatively.
(4) Prophylactic TXA group compared to control group.
(5) At least 1 of the outcome measures were included.

We had no restriction to route of administration, language of publication, or technique of AT.

Exclusion criteria consisted of studies including patients with blood dyscrasia or history of bleeding disorder.

### Study Selection and Data Extraction

Literature search results were exported to Covidence software, a web‐based collaboration software platform that streamlines the production of systematic and other literature reviews (Covidence systematic review software, Veritas Health Innovation, available at www.covidence.org). Prior to the formal screening process, a calibration exercise was undertaken to pilot and refine the screening process. Titles and abstracts were independently assessed by 2 reviewers (H.S. and P.C.) to identify all articles that met the inclusion criteria. Conflicts were resolved by discussion between the 2 authors. Two reviewers (H.S. and P.C) then independently assessed the full texts of articles to identify those that met all inclusion and exclusion criteria for the final analysis; any disagreements were resolved. Data were collected by 2 independent reviewers (H.S. and P.C) using a Microsoft Excel structured and pretested data collecting sheet. Data were compared between the 2 reviewers for accuracy.

### Data Items

Extracted data included author, year of publication, country and journal of publication, patients' demographics, procedures performed, number of surgeons involved, surgical technique. The other variables collected included number of patients treated with TXA, dosage, route of administration, perioperative blood volume loss, methods and techniques used to quantify intra‐ and postoperative blood loss (Table 1), number of patients with PTH, number and type of further interventions, duration of follow‐up.

**Table 1:** Characteristics of Included Trials

Reference (country)	Study design	N randomized	N (control/TXA)	Population/mean age, y	Surgical indication	Intervention/dosage	Procedure/technique	Outcome with available data
Falbe‐Hansen et al^13^ (Denmark)	RCT	1050	525/525	Adult/NR	NR	Topical application of TXA	Tonsillectomy/NR	Postop bleeding
Verstraete et al^14^ (Belgium)	RCT	82	45/37	Children/NR	NR	IV TXA, 20 mg/kg	AT/cold steel	Blood volume loss
Castelli and Vogt^15^ (Switzerland)	RCT	80	40/40	Adult/NR	NR	IV TXA, 500 mg	Tonsillectomy/NR	Blood volume loss
								Postop bleeding
								Need for intervention
								Adverse effects
George et al^16^ (India)	RCT	100	50/50	Adult and children/NR	Chronic tonsillitis	IV TXA, 10 mg/kg	Tonsillectomy/NR	Blood volume loss
								Postop bleeding
								Need for intervention
								Adverse effects
Brum et al^17^ (Brazil)	RCT	95	48/47	Children/6.88	OSAS	IV TXA, 10 mg/kg	AT/Cold Steel	Blood volume loss
								Postop bleeding
								Need for intervention
								Need for transfusion
								Adverse effects
Soliman et al^18^ (Saudi Arabia)	RCT	225	75/150	Children/7.08	Recurrent tonsillitis	IV TXA, 15 mg/kg	Tonsillectomy/Cold Steel or Hot technique (Bipolar)	Blood volume loss
								Postop bleeding
								Adverse effects
Santosh et al^19^ (India)	RCT	50	25/25	Children/14	Recurrent tonsillitis	IV TXA, 10 mg/kg	Tonsillectomy/Cold Steel	Blood volume loss
								Postop bleeding
								Need for intervention
								Need for transfusion
								Adverse effects
Elzayat and Elgebaly^20^ (Egypt)	RCT	100	50/50	Children/8.75	Recurrent tonsillitis	IV TXA, 15 mg/kg	Tonsillectomy/hot technique (bipolar)	Blood volume Loss
Fornazieri et al^21^ (Brazil)	RCT	63	32/31	Children/5.95	OSAS recurrent tonsillitis	IV TXA, 10 mg/kg	AT/cold steel	Blood volume loss
								Postop bleeding
								Need for intervention
								Adverse effects
Aboelsuod et al^22^ (Egypt)	RCT	82	41/41	Children/NR	OSAS	Topical application of TXA	Tonsillectomy/NR	Postop bleeding
								Need for intervention
								Adverse effects

Abbreviations: AT, adenotonsillectomy; IV, intravenous; NA, not applicable; NR, not reported; OSAS, obstructive sleep apnea syndrome; Postop, postoperative; RCT, randomized controlled trial; TXA, tranexamic acid.

### Outcome Measures

The main outcome of this SR is the risk of PTH. Secondary outcomes include volume of perioperative blood loss, need for transfusion, need for further intervention to address hemorrhage (medical and/or surgical), and the adverse effects of TXA.

### Risk of Bias in Individual Studies

The risk of bias for every study included in this SR and MA was assessed using the revised Cochrane risk of bias tool for randomized controlled trials (Risk of Bias 2 tool).^23^ Assessments were adjudicated independently by 2 reviewers (H.S, P.C.); disagreements were resolved. Studies were categorized as low, high, or unclear risk of bias based on the worst score obtained across the 6 domains.

### Certainty of Evidence and Strength of Recommendations

We evaluated the certainty of evidence and strength of recommendations using the Grading of Recommendations, Assessment, Development, and Evaluation (GRADE) system. The final quality of evidence was classified as high, moderate, low, or very low for each clinical outcome. Two reviewers (H.S. and P.C.) performed the classification of GRADE independently.^24^

### Data Synthesis

All statistical analyses were conducted using RevMan Web, version: 6.4.0. For continuous data (blood volume loss), a mean difference and 95% confidential interval (CI) were utilized. For outcomes that were dichotomous (PTH, need for transfusion, need for further intervention, and adverse effects), relative risk or risk ratio (RR) with its corresponding 95% CI. We used random‐effect models with inverse variance methods (Der Simonyan and Laird method).^25^ We also calculated the number needed to treat (NNT) with TXA to achieve 1 less PTH. The NNT was calculated using the formula: NNT = 1/[*π*_0_ × (1 − RR)] with an assumed comparator risk (*π*_0_) of 4.5%.^7^, ^26^, ^27^ Heterogeneity across individual studies was assessed using the *I*² index. Heterogeneity was considered significant for an *I*² value greater than 50%.^28^ We assessed publication bias visually using funnel plots. We conducted subgroup and sensitivity analyses based on predefined specified hypotheses that could explain potential statistical heterogeneity: risk of bias of trials, age of patients (adults vs children; an adult was defined as age ≥ 18 years old), indication for surgery, mode of administration of TXA, dose of TXA, and surgical technique.

## Results

### Literature Search

**Figure 1:**
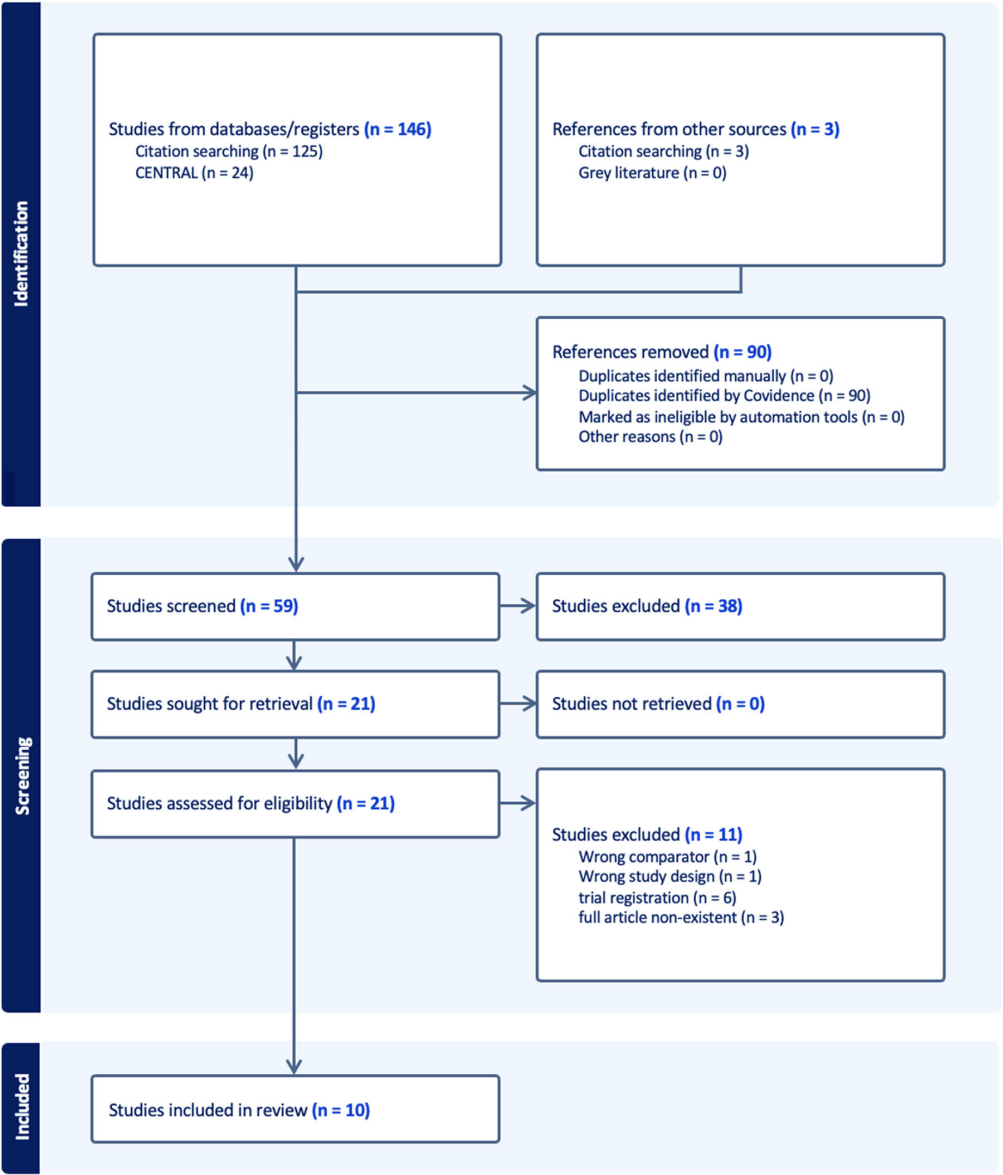
Preferred Reporting Items for Systematic Reviews and Meta‐analyses flowchart of search strategy for the present systematic review and meta‐analysis.

Our search strategy yielded 149 citations, from which 90 duplicates were removed. Twenty‐one articles were retrieved for full‐text screening. Ten articles were included in our quantitative analysis^13^, ^14^, ^15^, ^16^, ^17^, ^18^, ^19^, ^20^, ^21^, ^22^ (Figure 1).

### Characteristics of Trials

Characteristics of included trials are presented in Table 2. Three articles were conducted in Europe (Switzerland, Belgium, Denmark),^13^, ^14^, ^15^ 3 in Asia (India, Saudi Arabia),^16^, ^18^, ^19^ 2 in South America (Brazil),^23^, ^27^ and 2 in Africa (Egypt).^20^, ^22^ Seven articles included children, 2 articles included adult patients^13^, ^15^ and 1 article included both children and adults.^16^ Eight articles reported on intravenous (IV) prophylactic TXA at induction prior to tonsillectomy while the remaining 2 articles^13^, ^22^ reported on topical application of TXA after tonsillectomy. Seven articles included only tonsillectomy patients and 3 included patients undergoing AT.^14^, ^17^, ^21^

**Table 2:** Methods and Definitions Applied to Quantify Intraoperative Blood Volume Loss and PTH

Reference	Method used for quantifying intraoperative blood volume loss	Definition of PTH
Falbe‐Hansen et al^13^	Pre‐ and postoperative Hb and Hct Levels	Bleeding requiring treatment in the OR under general anesthesia
Verstraete et al^14^	Perdometer (A. B. Lars Ljungberg)	Outcome not measured
Castelli and Vogt^15^		Three‐point scale; 1—slight; 2—moderate; 3—severe
George et al^16^	Gravimetric method	Any bleeding regardless of severity or need for surgical intervention was reported
Brum et al^17^	contents of the aspiration flask	Presence of streaks of blood in the saliva or presence of live blood, recorded in the diary
Soliman et al^18^	Boezaart blood grading scale	ND
Santosh et al^19^	Gravimetric method and measurement of blood collected in the suction jar	ND
Elzayat and Elgebaly^20^	Measurement of blood collected in the suction jar Number and weight of bloody gauze Postoperative Hb and Hct Levels	Outcome not measured
Fornazieri et al^21^	Blood collected in the suction jar	Any bleeding regardless of severity or need for surgical intervention was reported
Aboelsuod et al^22^	Number of soaked cotton balls	Mild: no need for reoperation Severe: need for reoperation

Abbreviations: Hb, hemoglobin; Hct, hematocrit; ND, not defined; OR, operating room; PTH, posttonsillectomy hemorrhage.

### Qualitative Data Synthesis

The methods and techniques used to quantify intraoperative blood volume loss as well as the definition used in each RCT for PTH were summarized in Table 2.

### Quantitative Data Synthesis

#### PTH

Eight studies (n = 1627) reported on PTH.^13^, ^15^, ^16^, ^17^, ^18^, ^19^, ^21^, ^22^ A rate of 3.5% (n = 28) was observed in the TXA group compared to a rate of 6.2% (n = 52) in the control group.

**Figure 2:**
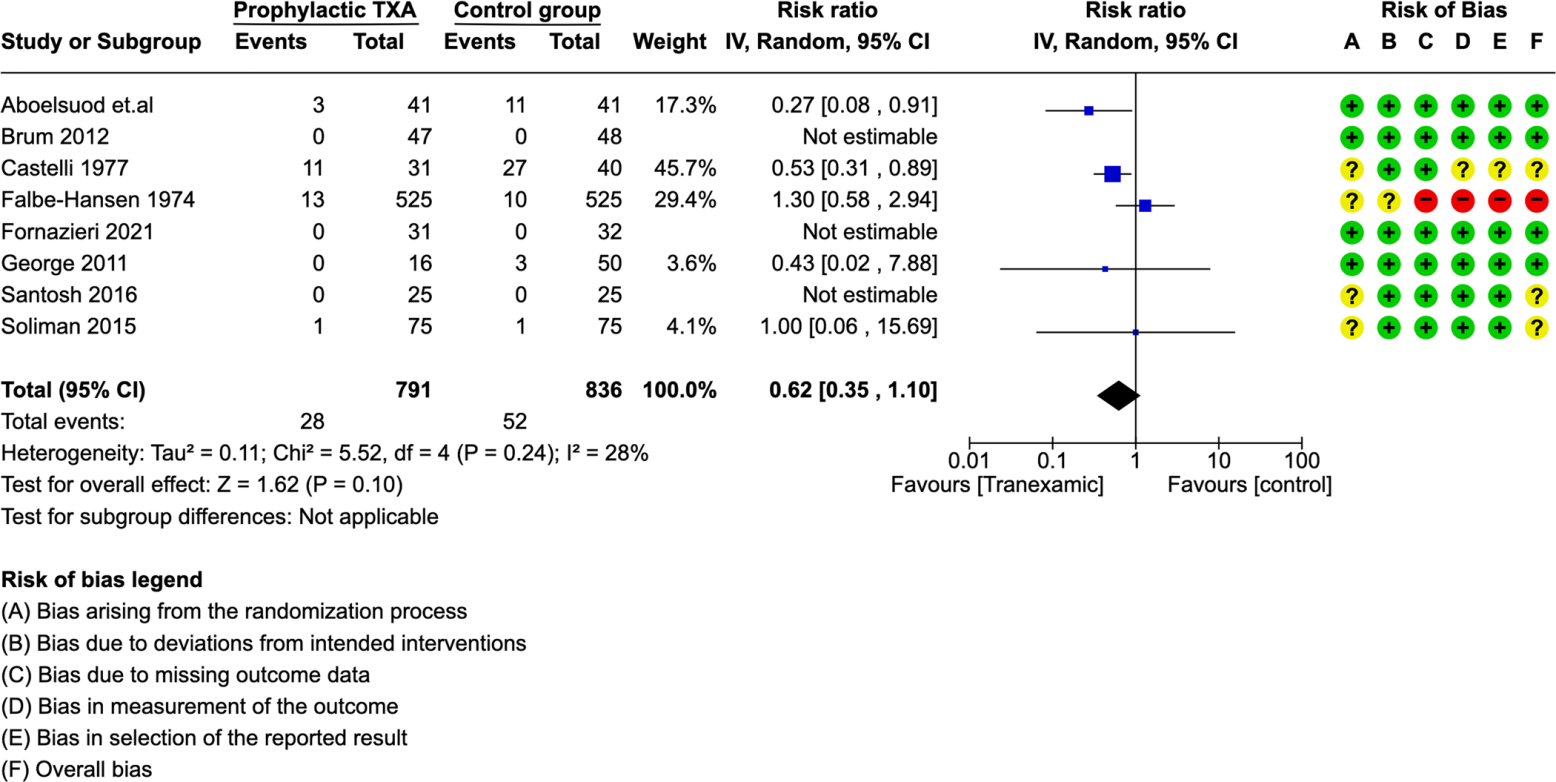
Forest plots showing effect of TXA on number of patients with PTH. CI, confidence interval; PTH, posttonsillectomy hemorrhage; TXA, tranexamic acid.

The MA showed a lower rate of hemorrhage in the TXA group (RR: 0.62 [0.35, 1.10], [*P* = .24]) (Figure 2). There was an overall low level of heterogeneity between studies illustrated by an *I*² value of 28%. Overall, the certainty of evidence for our primary outcome was moderate (Table 3).

**Table 3:** Certainty Assessment and Summary of Findings

	Certainty assessment						Events/number of patients				
	Number of studies	Study design	Risk of bias	Inconsistency	Indirectness	Imprecision	Other consideration	TXA	Control	RR (95% CI)	Certainty
PTH	8	RCT	NS	NS	NS	Serious^a^	None	28/791	52/836	0.62 (0.35‐1.10)	⊕⊕⊕ ○ moderate
Blood loss volume	8	RCT	NS	Serious^b^	Serious^c^	NS	None	N/A	N/A		⊕⊕ ○○ Low
Need for transfusion	2	RCT	NS	NS	NS	NS	None	0/72	0/73	Not estimable	⊕⊕⊕⊕ High
Need for further intervention	6	RCT	NS	NS	NS	Serious^d^	None	16/688	20/713	0.68 (0.23‐1.97)	⊕⊕⊕ ○ moderate
Adverse effects	7	RCT	NS	NS	NS	Serious^e^	None	3/310	0/311	7.00 (0.37‐131.28)	⊕⊕⊕ ○ moderate

Abbreviations: CI, confidence interval; N/A, not applicable; NS, not serious; PTH, posttonsillectomy hemorrhage; RCT, randomized controlled trials; RR, relative risk; TXA, tranexamic acid.

^a^ Overall risk ratio overlapped no effect (RR = 0.62 [0.35‐1.10]).

^b^ Substantial heterogeneity among included studies (*I*² = 98%).

^c^ Studies used different methods to quantify blood loss.

^d^ Overall risk ratio overlapped no effect (RR = 0.68 [0.23‐1.97]).

^e^ Overall risk ratio overlapped no effect (RR = 7.00 [0.37‐131.28]).

**Figure 3:**
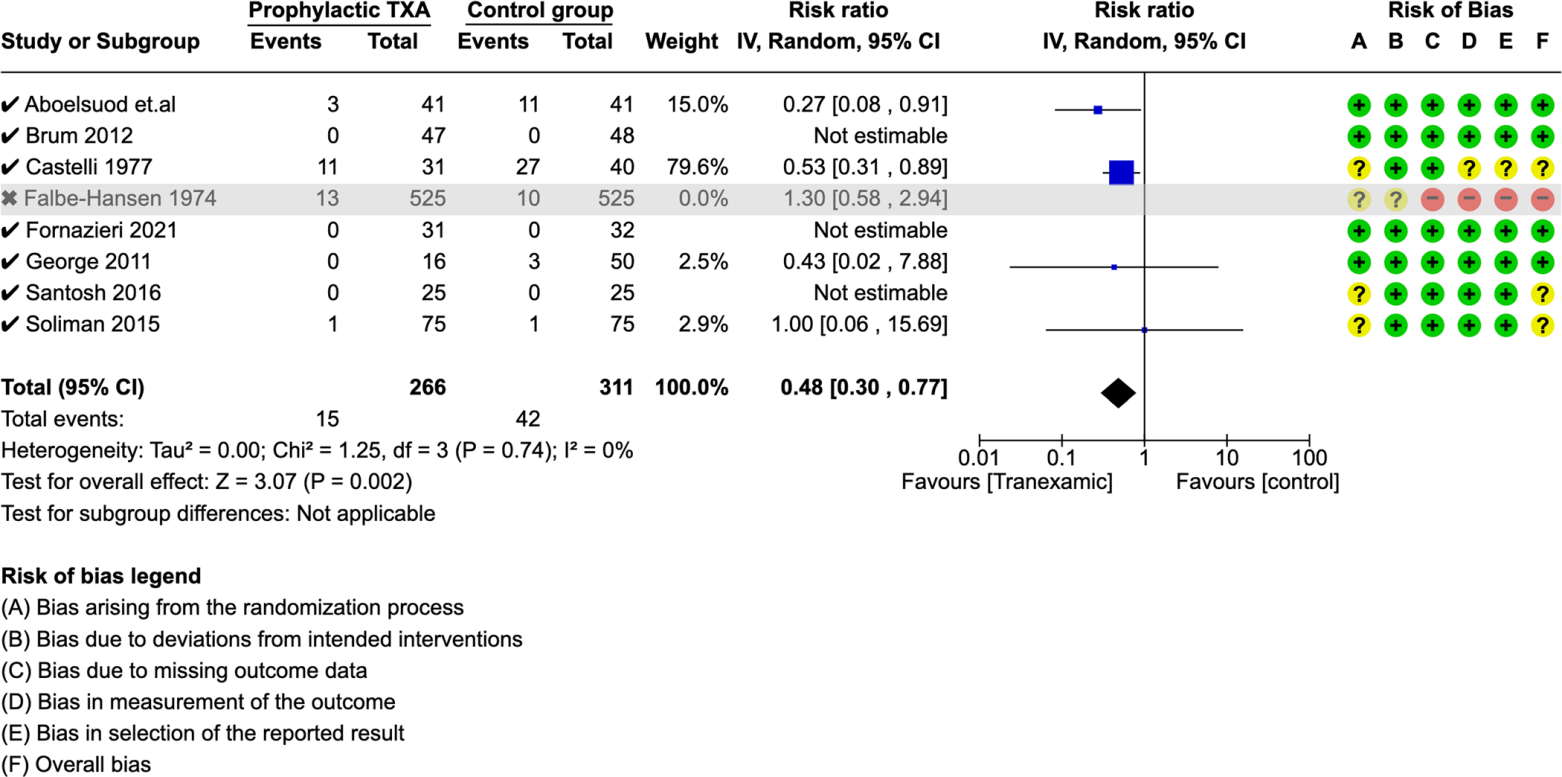
Sensitivity analysis—PTH. CI, confidence interval; PTH, posttonsillectomy hemorrhage; TXA, tranexamic acid.

A sensitivity analysis was performed by excluding 1 study with high‐risk of bias.^13^ This showed a significant lower rate of PTH in the TXA group (RR: 0.48 [0.30, 0.77]). The heterogeneity across remaining studies was *I*² = 0%, (Figure 3).

##### Subgroup Analysis: Risk of Bias

**Figure 4:**
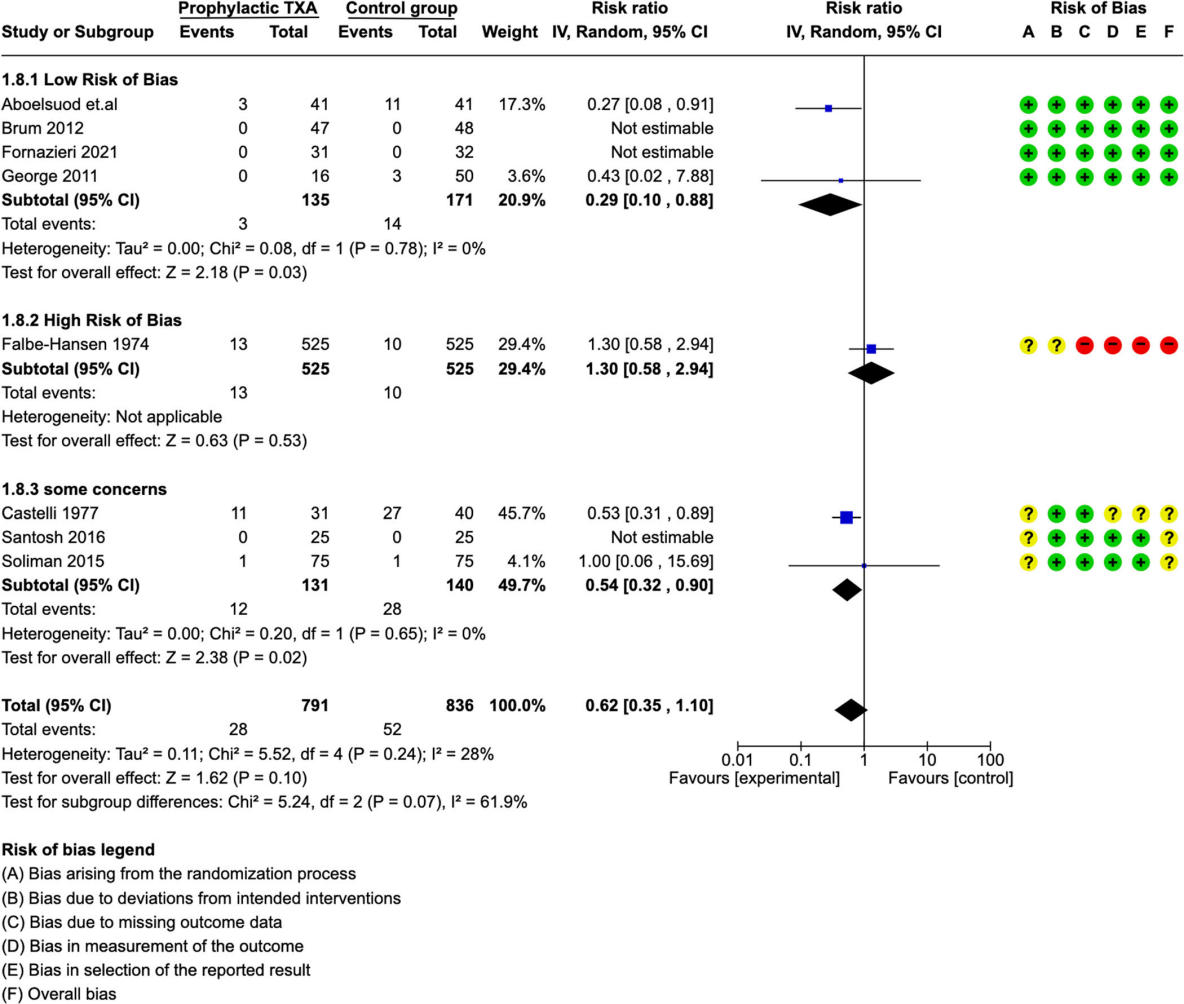
Subgroup analysis: Forest plots showing effect of TXA on number of patients with PTH—risk of bias. CI, confidence interval; PTH, posttonsillectomy hemorrhage; TXA, tranexamic acid.

Subgroup analyses stratified by risk of bias was illustrated in (Figure 4). It showed a significantly lower rate of PTH compared to control in group with low risk of bias studies (RR: 0.29 [0.10, 0.88]). There was no evidence of heterogeneity between low risk of bias studies (*I*² = 0%).

##### Subgroup Analysis: Route of Administration

**Figure 5:**
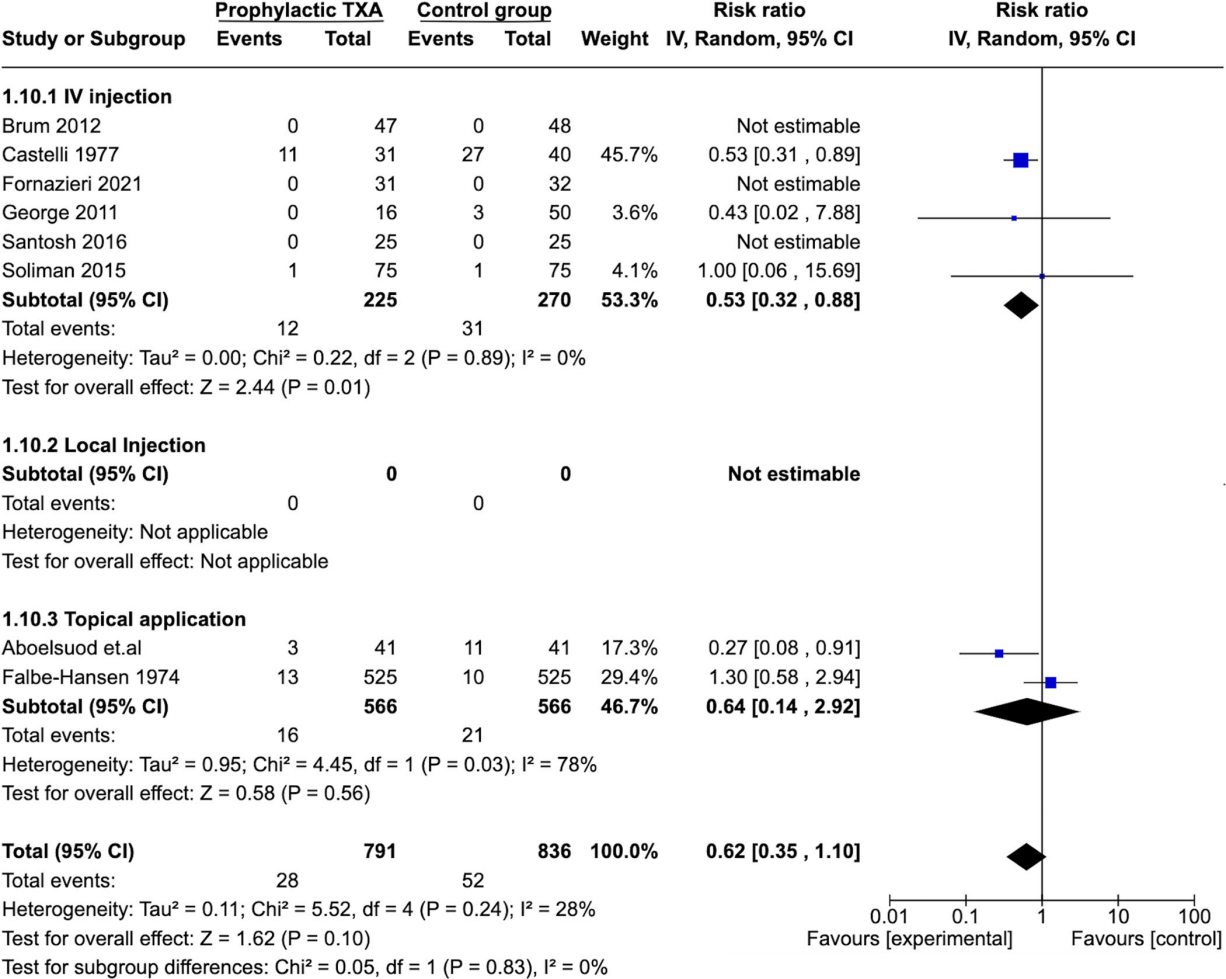
Subgroup analysis: PTH—mode of administration. CI, confidence interval; PTH, posttonsillectomy hemorrhage; TXA, tranexamic acid.

Subgroup analysis was performed according to the route of administration (Figure 5). When TXA is given intravenously (6 studies),^15^, ^16^, ^17^, ^18^, ^19^, ^21^ there was a significantly lower risk of PTH compared to control group (RR: 0.32 [0.13, 0.77]). The heterogeneity index value is 0%. Two studies evaluated TXA given topically.^13^, ^22^ This showed a nonsignificant reduction in PTH rate (RR: 0.80 [0.39, 1.63]). There was a high heterogeneity between the studies (*I*² = 80%).

##### Subgroup Analysis: Adults (Age ≥18 years) Versus Children (<18 Years)

**Figure 6:**
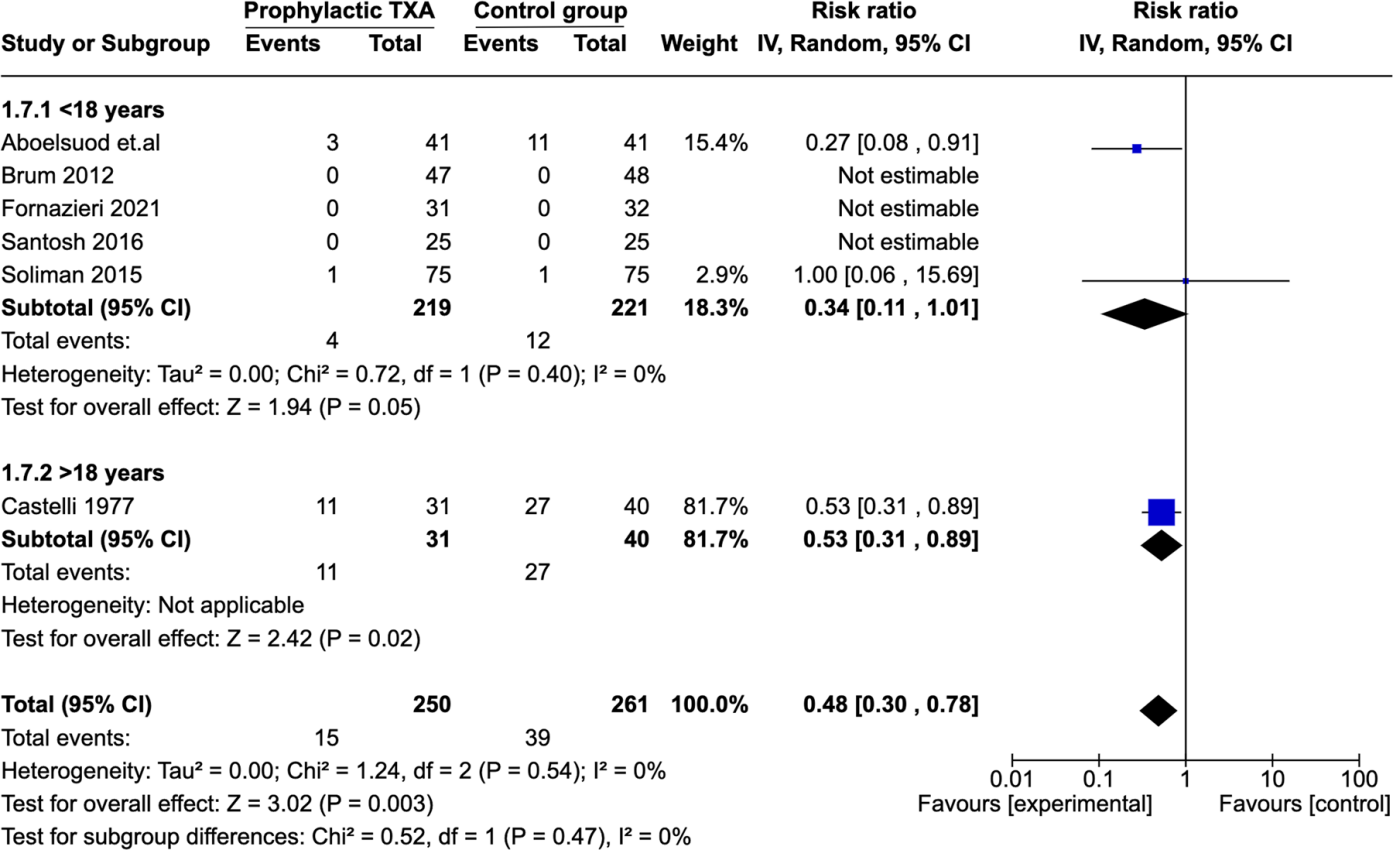
Subgroup analysis: PTH—adult versus pediatric. CI, confidence interval; PTH, posttonsillectomy hemorrhage; TXA, tranexamic acid.

Subgroup analysis was performed according to age (Figure 6). Five studies reported on PTH in children.^17^, ^18^, ^19^, ^21^, ^22^ In 3 studies, the relative risk was not estimable as no events were recorded in both groups. The results showed a non‐significant decrease in PTH risk in favor of the TXA group (RR: 0.34 [0.11, 1.01]) in the children category. The heterogeneity index value across studies was 0%. Only 1 study reported on adult patients (age ≥ 18 years).^15^ It showed a significantly lower RR of PTH (RR: 0.53 [0.31, 0.89]) in favor of the TXA group.

##### Subgroup Aanalysis: Indication for Surgery (Recurrent/Chronic Tonsillitis vs OSAS)

**Figure 7:**
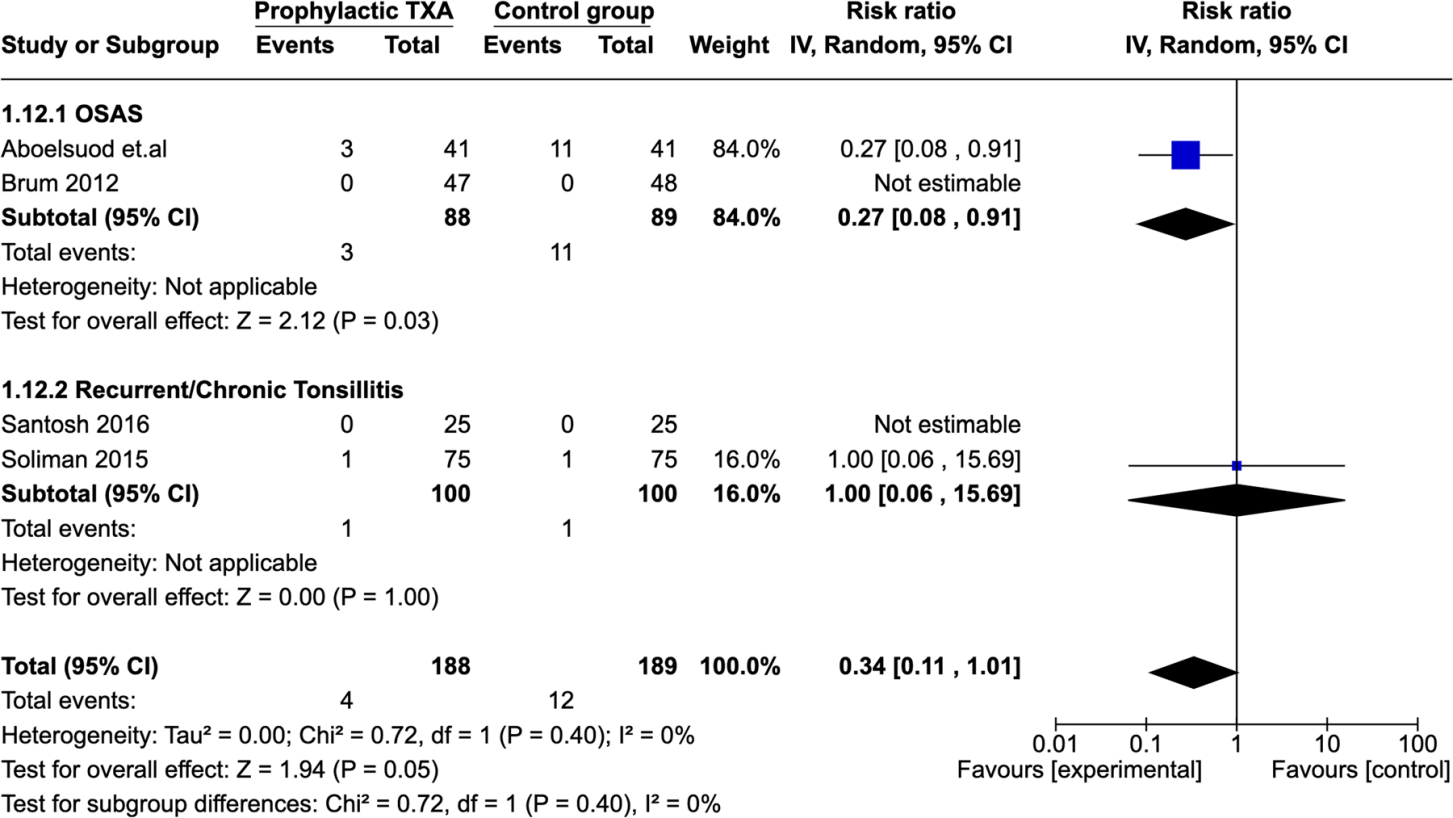
Subgroup analysis: Indication for surgery (recurrent/chronic tonsillitis vs OSAS). CI, confidence interval; OSAS, obstructive sleep apnea syndrome; TXA, tranexamic acid.

Subgroup analysis was performed according to surgical indication (Figure 7). Two studies reported on PTH in patient with OSAS.^17^, ^22^ The results showed a significant reduction in the PTH rate for the TXA group (RR: 0.27 [0.08, 0.91]). In contrast, 2 studies reported on patients with recurrent or chronic tonsillitis^18^, ^19^ and did not find a reduction in PTH rate for the TXA group (RR: 1.00 [0.06, 15.69]).

##### Subgroup Analysis: Dose of IV TXA (≤10 vs >10 mg/kg)

**Figure 8:**
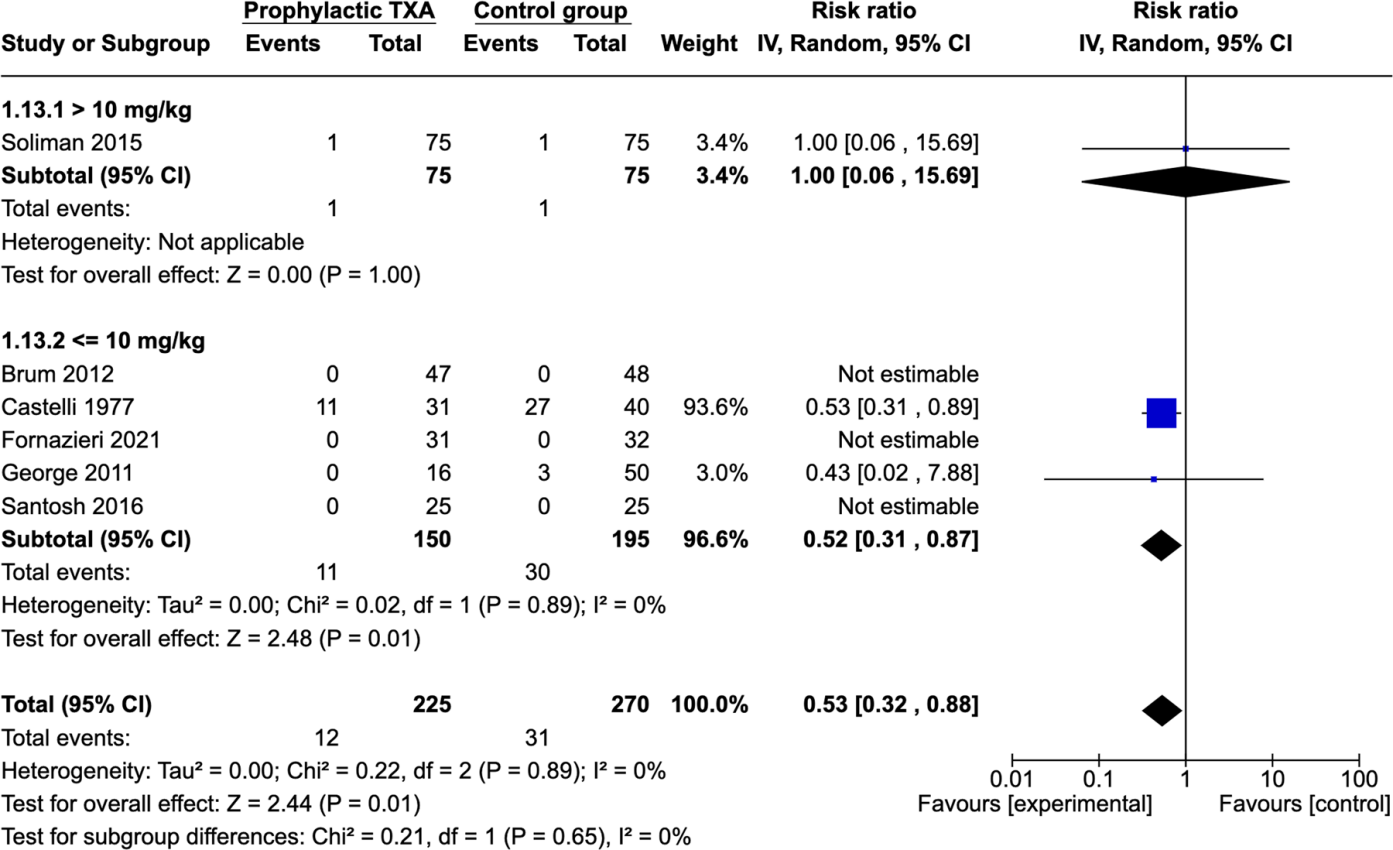
Subgroup analysis: dose of IV TXA (≤10 vs >10 mg/kg). CI, confidence interval; IV, intravenous; TXA, tranexamic acid.

Subgroup analysis was performed according to the TXA dose (Figure 8). Five studies reported on PTH in patients receiving IV TXA (≤10 mg/kg).^15^, ^16^, ^17^, ^19^, ^21^ Overall, there was a significant decrease in PTH rate (RR: 0.52 [0.31; 0.87]). The heterogeneity index value across studies was 0%. Only 1 study reported on PTH in patients receiving IV TXA (>10 mg/kg).^18^ There was no decrease in PTH rate (RR: 1.00 [0.06; 15.69]).

### Blood Volume Loss

**Figure 9:**
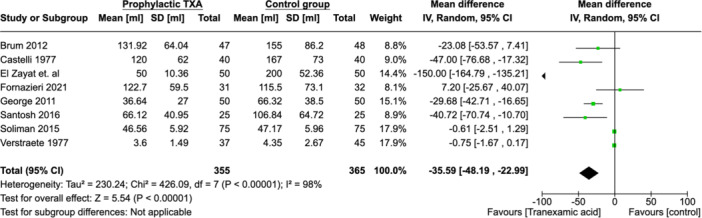
Forest plots showing effect of TXA on intraoperative blood loss volume. CI, confidence interval; TXA, tranexamic acid.

Eight studies (n = 720) reported on intraoperative blood loss.^14^, ^15^, ^16^, ^17^, ^18^, ^19^, ^20^, ^21^ There was significant heterogeneity among studies (*χ* ^2^ = 426.09, *df* = 7 (*P* < .00001); *I*² = 98%). The MA showed a significant decrease in intraoperative blood loss by 35.59 mL (−48.19, −22.99) (Figure 9). Publication biases were assessed using funnel plot (Supplemental Figure S1, available online).

### Need for Further Intervention

**Figure 10:**
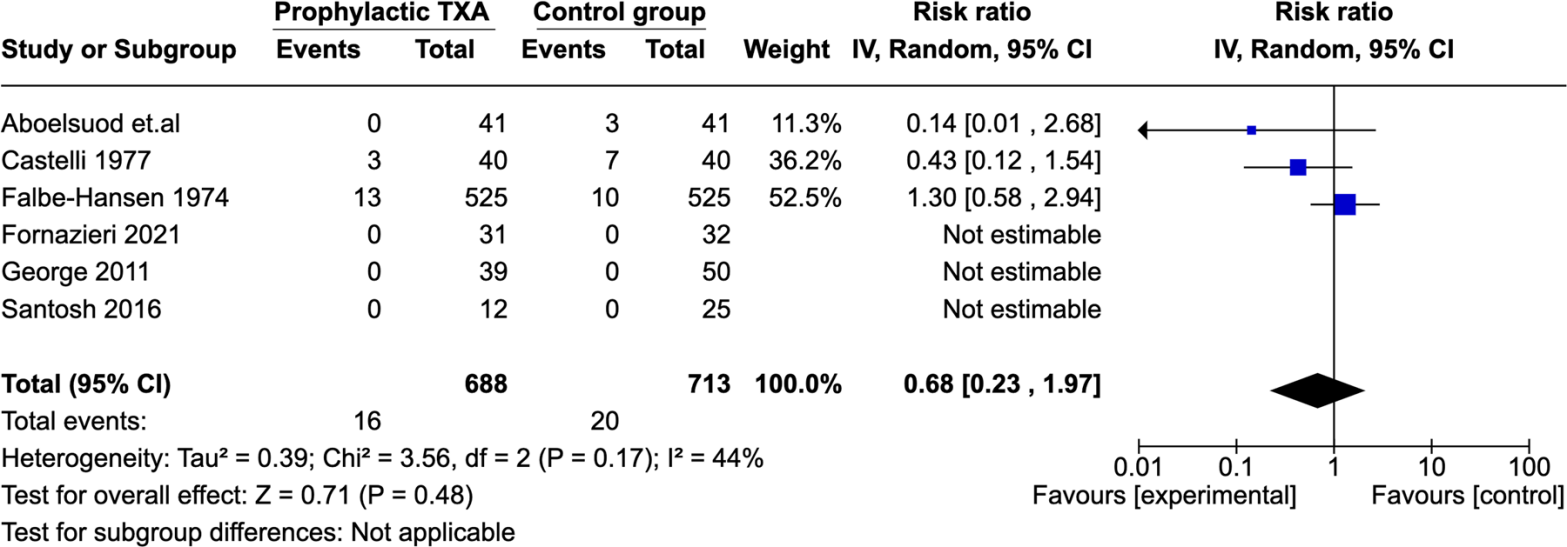
Forest plot showing effect of TXA on number of patients required further intervention. CI, confidence interval; TXA, tranexamic acid.

Six studies reported on the need for further intervention (n = 1401).^13^, ^15^, ^16^, ^19^, ^21^, ^22^ There was no significant difference between the 2 groups as illustrated in (Figure 10). There was a moderate level heterogeneity across the studies reflected by an *I*² value of 44%. One study, showed particularly high rate of intervention among TXA group.^13^

### Need for Transfusion

Only 2 studies reported on the need for transfusion.^17^, ^19^ There were no reported cases of patients requiring transfusion in both studies.

### Adverse Effects

Seven studies reported on Adverse effects.^15^, ^16^, ^17^, ^18^, ^19^, ^21^, ^22^ Six studies reported no adverse effects; only 1 study reported 3 minor side effects in the TXA group (1 patient had dizziness, 1 had mild headache, and 1 experienced a single episode of postoperative vomiting).^15^ No major adverse effects were reported across all studies.

### NNT

The computation of the NNT with TXA to achieve 1 less PTH using a RR of 0.48 lead to a NNT equal to 43.

## Discussion

PTH is a serious complication. It requires mobilization of resources, admission to hospital stay, surveillance in step up units, transfusion, and possibly reintervention in the operating room (OR).^29^ It is a stressful experience for the patient, the family and the medical team. Efforts have been made to address this complication by the introduction of new surgical techniques such as intracapsular tonsillectomy.^30^ However, these surgical techniques (eg, coblator or microdebrider intracapsular tonsillectomy) are yet to be the standard of care, are not widely available and affordable, and carry some drawbacks such as the risk of residual tonsillar tissue and the risk of tonsillar regrowth.^31^

Our SR is not the first of its kind to address the role of TXA in tonsillectomy. However, we carried this SR to (1) support previous observations considering new emerging data, (2) address some methodological gaps in previous SR, and (3) generate a MA based only on high quality evidence (RCTs) to support or argue against regular use of TXA in tonsillectomy.

Our SR showed a lower risk of PTH after tonsillectomy with prophylactic TXA administration; this risk reduction was statistically significant after exclusion of 1 study with high‐risk of bias.^13^ This observation is consistent with 2 previous SR published on the topic in 2013^32^ (0.51 [0.25‐1.07]) and 2022^2^ (RR: 0.42 [0.28‐0.65]). Our study also showed that in comparison to IV TXA injection (RR: 0.53 [0.32‐0.88]), topical application was not as effective (RR: 0.62 [0.35‐1.10]). This distinction in efficacy (IV vs topical) was not previously reported.

We were particularly interested in this SR, to test the efficacy of TXA on PTH rate in different age groups. A subgroup analysis was carried out for adults (>18 years) versus children (≤18 years).

The PTH rate in the TXA group was low in both adult and pediatric population, although it only reaches statistical significance in the adult sub‐group. Those findings are consistent with Kuo et al MA.^2^ However, this appears counterintuitive considering that adult tonsillectomy carries a higher risk of bleeding.^33^ One possible explanation for these findings could be the age threshold (18 years) we applied in this MA, which may be more of a legal demarcation than a functional one. Some pediatric patients in the preadulthood or adolescent category (ages 12‐18) demonstrate indications for tonsillectomy that are similar to those seen in adult patients (tonsillitis is more often an indication for tonsillectomy in older children than younger children).^34^ A different way to look at the problem is by a subgroup analysis based on indication rather than age. Our findings showed a significant decrease in PTH rate in TXA group when tonsillectomy is performed for OSAS versus recurrent/chronic tonsillitis. Those findings were not reported in the previous MA.

In this SR, we opted to choose PTH as our primary outcome in comparison to intraoperative blood volume loss as per the 2 previous SR.^2^, ^32^ From a practical and clinical standpoint, PTH is more relevant to the clinician. Intraoperative bleeding is usually a technical challenge addressed with conventional technique to stop the bleeder (eg, bipolar diathermy, ligature, pressure), except for some rare instances where it can be life threatening.^5^

The NNT is a statistical inference of the size of therapeutic effect one might expect in clinical practice. The NNT of 43 predicts that routine use of a prophylactic IV dose of TXA in 43 patients would result in 1 less patient having PTH. It's important to note the *π*_0_ was set to 4.5% in this study which is the general accepted rate of PTH in the general population. Some studies however reported a much higher rates of PTH reaching 10.8%.^33^ Altogether, taking into consideration how common tonsillectomy is performed in the general otolaryngology practice and the harmful consequences associated with PTH, we believe that the NNT in this case reflect a therapeutic benefit and a clinically relevant effect size.^35^

**Figure 11:**
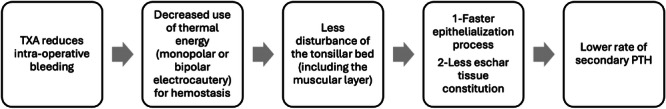
Diagram illustrating pathophysiological impact of TXA on secondary PTH. PTH, posttonsillectomy hemorrhage; TXA, tranexamic acid.

An important question that wasn't specifically addressed and answered in this SR is whether TXA impacts primary or secondary hemorrhage. From a basic pharmacology standpoint, TXA is a synthetic analogue to the amino acid lysine.^36^ it promotes antifibrinolysis by competitively binding to the lysine‐binding site on plasmin and plasminogen and precludes subsequent fibrin clot degradation by plasmin. It seems reasonable then to believe that TXA impacts mainly primary PTH by the preservation of fibrin clot and prevention of its early degradation.^36^ We hypothesize however that TXA may also reduce secondary PTH by reducing perioperative bleeding and oozing. The proposed pathophysiological mechanism of action is illustrated in Figure 11. This hypothesis remains to be verified in future works.

### Strength and Weakness

Our SR has several strengths. We opted for a strong design including only RCTs to generate high quality evidence. Our primary outcome was chosen based on practical considerations and intentions with the endpoint a reduction in tonsillectomy‐related morbidity. We performed subgroup and sensitivity analyses to overcome heterogeneity across included studies and to explore the impact of TXA among special group of interest (eg adult vs pediatric). We also evaluated the certainty of evidence and strength of recommendations using the GRADE system. We performed a qualitative data analysis to evaluate the definitions for PTH used in each of the included RCT. Falbe‐Hansen et al defined PTH as bleeding requiring treatment in the OR.^13^ This study was subsequently excluded in the sensitivity analysis we performed. We believe that a broad and inclusive definition for PTH is more appropriate.^37^ Any type of PTH needs to be reported, whether it is simply blood‐tinged saliva, or bleeding that requires intervention in the OR. PTH can be distressing for patients regardless of its severity. Patients have no direct way of visualizing or controlling their bleeding. Bleeding may be recurring and minor bleeding can be the first sign (sentinel bleed) of more significant, life‐threatening bleeding.^38^ Some patients, particularly children, may swallow or aspirate blood rather than spitting it out.^39^ Therefore, it is crucial to investigate all cases of PTH.

Our study has some limitations. First, the difficulty in extracting data and combining results from trials that utilize different surgical techniques, TXA dosage and routes of administration, techniques of blood volume loss reporting, and definitions used for PTH. We performed multiple subgroups analysis to address heterogeneity across studies. However, the results of those analyses should be interpreted cautiously due to lack of statistical power which could increase the risk of reporting false‐positive results.^40^, ^41^ Our study did not take into consideration, changes in practice and basic research methodology for RCT execution and documentation over time; old studies were still included in this SR provided they are RCT. One study published in 1974^13^ with high risk of bias may had some impact on our MA results and our confidence and certainty in the evidence we provided.

## Conclusion

This SR showed that prophylactic TXA is associated with a reduction in PTH rate. Those results are however significant only when studies with high risk of bias are excluded. More studies with high quality design are still needed to support our conclusion.

## Author Contributions

**Hussein Smaily**, contributed to the design, conduct, and drafting of the manuscript; **Patrick Cherfane**, contributed to the conduct and drafting of the manuscript.

## Disclosures

### Competing interests

The authors have no conflicts of interest to disclose.

### Funding source

The authors have no funding or financial relationships.

## Supporting information

Supporting information.
