# Supplementary material for: The Utility and Safety of Prophylactic Tranexamic Acid in Tonsillectomy: A Systematic Review and Meta‐analysis
Source: Otolaryngol Head Neck Surg. 2024 Oct 1;172(1):36–49. doi: 10.1002/ohn.973 (PMC11697522; doi:10.1002/ohn.973)
Supplement: Supplementary file 1 — Supporting information. [file OHN-172-36-s001.docx]

Supplemental Materiel – Table of content

Supplemental Figure 1. Funnel plot for post-operative hemorrhage

Supplemental Table 1: Medline search strategy

Supplemental Figure 1: Funnel plot for post-operative hemorrhage.


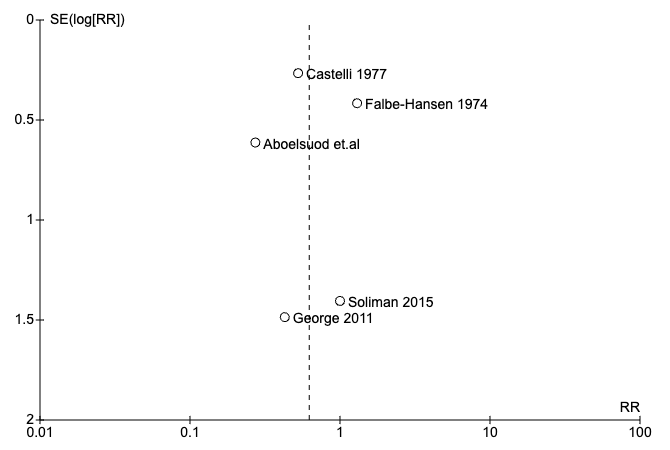


Table 1: Medline search strategy

| **Search number** | **Query** | **Sort By** | **Filters** | **Search Details** | **Results** | **Time** |
| --- | --- | --- | --- | --- | --- | --- |
| **27** | #15 AND #26 |  |  | ("Tonsillectomy"[Title/Abstract] OR "tonsil*"[Title/Abstract] OR "amygdalectomy"[Title/Abstract] OR "adenotonsillectomy"[Title/Abstract] OR "tonsillotomy"[Title/Abstract] OR "adeno-tonsillectomy"[Title/Abstract] OR "Tonsillectomy"[MeSH Terms]) AND ("tranexamic acid"[MeSH Terms] OR "tranexamic acid"[Title/Abstract] OR "acide tranexamique"[Title/Abstract] OR "cyclokapron"[Title/Abstract] OR "tranex*"[Title/Abstract]) AND (("randomized controlled trial"[Publication Type] OR "controlled clinical trial"[Publication Type] OR "randomized"[Title/Abstract] OR "placebo"[Title/Abstract] OR "drug therapy"[MeSH Subheading] OR "randomly"[Title/Abstract] OR "trial"[Title/Abstract] OR "groups"[Title/Abstract]) NOT (("animals"[MeSH Terms:noexp] OR "animals"[All Fields]) NOT "humans"[MeSH Terms])) | 23 | 19:22:16 |
| **26** | #24 NOT #25 |  |  | ("randomized controlled trial"[Publication Type] OR "controlled clinical trial"[Publication Type] OR "randomized"[Title/Abstract] OR "placebo"[Title/Abstract] OR "drug therapy"[MeSH Subheading] OR "randomly"[Title/Abstract] OR "trial"[Title/Abstract] OR "groups"[Title/Abstract]) NOT (("animals"[MeSH Terms:noexp] OR "animals"[All Fields]) NOT "humans"[MeSH Terms]) | 5,126,289 | 19:21:56 |
| **25** | animals NOT Humans[MeSH Terms] |  |  | ("animals"[MeSH Terms:noexp] OR "animals"[All Fields]) NOT "humans"[MeSH Terms] | 5,235,676 | 19:21:14 |
| **24** | #16 OR #17 OR #18 OR #19 OR #20 OR #21 OR #22 OR #23 |  |  | "randomized controlled trial"[Publication Type] OR "controlled clinical trial"[Publication Type] OR "randomized"[Title/Abstract] OR "placebo"[Title/Abstract] OR "drug therapy"[MeSH Subheading] OR "randomly"[Title/Abstract] OR "trial"[Title/Abstract] OR "groups"[Title/Abstract] | 5,884,885 | 19:18:42 |
| **23** | groups[Title/Abstract] |  |  | "groups"[Title/Abstract] | 2,629,073 | 19:17:14 |
| **22** | trial[Title/Abstract] |  |  | "trial"[Title/Abstract] | 791,134 | 19:17:02 |
| **21** | randomly[Title/Abstract] |  |  | "randomly"[Title/Abstract] | 421,632 | 19:16:00 |
| **20** | Drug Therapy[MeSH Subheading] |  |  | "drug therapy"[MeSH Subheading] | 2,638,390 | 19:15:40 |
| **19** | placebo[Title/Abstract] |  |  | "placebo"[Title/Abstract] | 250,137 | 19:14:51 |
| **18** | randomized[Title/Abstract] |  |  | "randomized"[Title/Abstract] | 682,028 | 19:14:36 |
| **17** | controlled clinical trial[Publication Type] |  |  | "controlled clinical trial"[Publication Type] | 695,046 | 19:14:17 |
| **16** | randomized controlled trial[Publication Type] |  |  | "randomized controlled trial"[Publication Type] | 604,504 | 19:13:12 |
| **15** | #13 AND #14 |  |  | ("Tonsillectomy"[Title/Abstract] OR "tonsil*"[Title/Abstract] OR "amygdalectomy"[Title/Abstract] OR "adenotonsillectomy"[Title/Abstract] OR "tonsillotomy"[Title/Abstract] OR "adeno-tonsillectomy"[Title/Abstract] OR "Tonsillectomy"[MeSH Terms]) AND ("tranexamic acid"[MeSH Terms] OR "tranexamic acid"[Title/Abstract] OR "acide tranexamique"[Title/Abstract] OR "cyclokapron"[Title/Abstract] OR "tranex*"[Title/Abstract]) | 44 | 19:04:06 |
| **14** | #8 OR #9 OR #10 OR #11 OR #12 |  |  | "tranexamic acid"[MeSH Terms] OR "tranexamic acid"[Title/Abstract] OR "acide tranexamique"[Title/Abstract] OR "cyclokapron"[Title/Abstract] OR "tranex*"[Title/Abstract] | 7,517 | 19:03:54 |
| **13** | #1 OR #3 OR #4 OR #5 OR #6 OR #7 |  |  | "Tonsillectomy"[Title/Abstract] OR "tonsil*"[Title/Abstract] OR "amygdalectomy"[Title/Abstract] OR "adenotonsillectomy"[Title/Abstract] OR "tonsillotomy"[Title/Abstract] OR "adeno-tonsillectomy"[Title/Abstract] OR "Tonsillectomy"[MeSH Terms] | 36,566 | 19:03:19 |
| **12** | tranex*[Title/Abstract] |  |  | "tranex*"[Title/Abstract] | 6,696 | 19:00:37 |
| **11** | cyclokapron[Title/Abstract] |  |  | "cyclokapron"[Title/Abstract] | 11 | 19:00:30 |
| **10** | acide tranexamique[Title/Abstract] |  |  | "acide tranexamique"[Title/Abstract] | 13 | 19:00:22 |
| **9** | tranexamic acid[Title/Abstract] |  |  | "tranexamic acid"[Title/Abstract] | 6,648 | 19:00:11 |
| **8** | tranexamic acid[MeSH Terms] |  |  | "tranexamic acid"[MeSH Terms] | 5,035 | 18:59:54 |
| **7** | tonsillectomy[MeSH Terms] |  |  | "tonsillectomy"[MeSH Terms] | 10,915 | 18:57:34 |
| **6** | adeno-tonsillectomy[Title/Abstract] |  |  | "adeno-tonsillectomy"[Title/Abstract] | 189 | 18:56:54 |
| **5** | tonsillotomy[Title/Abstract] |  |  | "tonsillotomy"[Title/Abstract] | 289 | 18:56:43 |
| **4** | adenotonsillectomy[Title/Abstract] |  |  | "adenotonsillectomy"[Title/Abstract] | 2,984 | 18:56:28 |
| **3** | amygdalectomy[Title/Abstract] |  |  | "amygdalectomy"[Title/Abstract] | 151 | 18:55:47 |
| **1** | (Tonsillectomy[Title/Abstract]) OR (tonsil*[Title/Abstract]) |  |  | "Tonsillectomy"[Title/Abstract] OR "tonsil*"[Title/Abstract] | 33,572 | 18:54:49 |
